# Endurance exercise under short‐duration intermittent hypoxia promotes endurance performance via improving muscle metabolic properties in mice

**DOI:** 10.14814/phy2.15534

**Published:** 2022-12-14

**Authors:** Junichi Suzuki

**Affiliations:** ^1^ Laboratory of Exercise Physiology, Health and Sports Sciences, Course of Sports Education, Department of Education Hokkaido University of Education Iwamizawa Japan

**Keywords:** Bayesian data analysis, heat shock factor 1, intermittent hypoxia, monocarboxylate transporters, NT‐PGC1alpha

## Abstract

This study was designed to (1) investigate the effects of acute exercise under intermittent hypoxia on muscle mRNA and protein levels, and (2) clarify the mechanisms by which exercise under intermittent hypoxia improves endurance capacity. Experiment‐1: Male mice were subjected to either acute endurance exercise, exercise under hypoxia (14% O_2_), exercise under intermittent hypoxia (Int, three cycles of room air [10 min] and 14% O_2_ [15 min]). At 3 h after exercise under intermittent hypoxia, sirtuin‐6 mRNA levels and nuclear prolyl hydroxylases‐2 protein levels were significantly upregulated in white gastrocnemius muscle in the Int group. Experiment‐2: Mice were assigned to sedentary control (Sed), normoxic exercise‐trained (ET), hypoxic exercise‐trained (HYP) or exercise‐trained under intermittent hypoxia (INT) groups. Exercise capacity was significantly greater in the INT group than in the ET and HYP group. Activity levels of citrate synthase were significantly greater in the INT group than in the HYP group in soleus (SOL) and red gastrocnemius muscles. In SOL, nuclear N‐terminal PGC1α levels were considerably increased by the INT training (95% confidence interval [CI]: 1.09–1.79). The INT significantly increased pyruvate dehydrogenase complex activity levels in left ventricle (LV). Monocarboxylate transporter‐4 protein levels were significantly increased after the INT training in LV. Capillary‐to‐fiber ratio values were significantly increased in SOL and were substantially increased in LV (CI: 1.10–1.22) after the INT training. These results suggest that exercise training under intermittent hypoxia represents a beneficial strategy for increasing endurance performance via improving metabolic properties and capillary profiles in several hind‐leg muscles and the heart.

## Graphical abstract

Exercise training under intermittent hypoxia represents a beneficial strategy for increasing endurance performance via improving metabolic properties and capillary profiles in hind‐leg muscles and the heart.

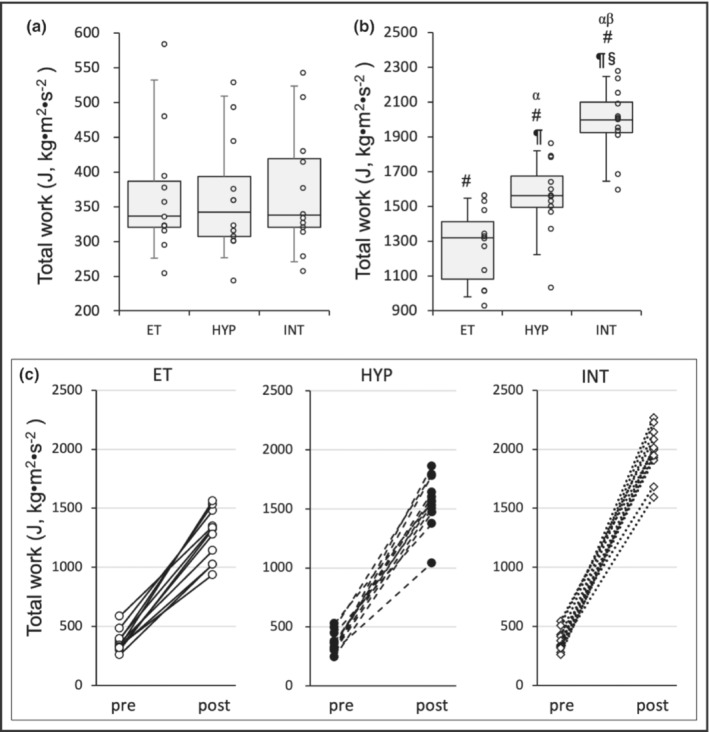

## 1 INTRODUCTION

To enhance exercise performance, exercise training under hypoxia was proposed in the early 1990s. Several forms of training procedures have been developed to improve athletic performance at sea level or high altitude, e. g. “live low‐train high”, and “live high‐train low.” Although many experimental procedures have been investigated, an essential strategy to improve athletic performance, especially endurance capacity at sea level, has not been clearly established (Terrados et al., 1990; Vogt & Hoppeler, 2010).

Short‐duration intermittent hypoxia (12% O_2_ [15 min], room air [10 min], 4 cycles per day) followed by running exercise in normoxia for 3 weeks markedly improved exercise performance by enhancing total carnitine palmitoyl transferase (CPT) activity and mRNA expression of peroxisome proliferator‐activated receptor gamma coactivator 1‐alpha (PGC‐1α) in mice (Suzuki, 2016). Moreover, using well‐trained mice, this experimental procedure was shown to improve endurance capacity via improving fatty acid and pyruvate oxidation in hind‐leg muscles (Suzuki, 2019). In these studies, daily normoxic exercise and intermittent hypoxic exposure were performed separately. It raised a hypothesis that doing exercise simultaneously with intermittent hypoxic exposure may improve endurance capacity effectively. If so, a less time‐consuming strategy could be provided to athletes. Thus, the present study was sought to examine acute response of exercise under intermittent hypoxia on gene expression and protein levels and chronic effects of these procedure on endurance performance and muscle metabolism.

Chronic hypoxic exposure has shown to increase glycolytic enzyme activity and, in contrast, to decrease lipid oxidation (Kennedy et al., 2001) probably for surviving at low oxygen atmosphere. Under hypoxic conditions, hypoxia inducible factor (HIF) is stabilized and translocated to the nucleus, thereby upregulating HIF responsive genes (Maxwell et al., 1999). Chronic stabilization of HIF suppressed fatty acid oxidation by downregulating peroxisome proliferator‐activated receptor α (PPARα; Belanger et al., 2007) and PGC‐1α levels in vitro (Slot et al., 2014). This may be one of the reasons why controversial results has been reported concerning effects of exercise training using hypoxic atmosphere on endurance performance.

Reduction in HIF levels, thus, promotes oxidative metabolism in skeletal muscles thereby enhancing endurance performance. HIF protein levels are regulated by its target prolyl hydroxylases (PHD 1–3; Bruick & McKnight, 2001). PHDs catalyze HIF hydroxylation, in these PHD2 was shown to play the primary role (Berra et al., 2003). If hypoxic stimuli transiently stabilize HIF, its target PHD2 degrades HIF as a negative‐feedback. Factor‐inhibiting HIF (FIH) was shown to suppress transcriptional activity of HIF target genes via inhibiting binding to the coactivator CBP/p300 (Mahon et al., 2001). Sirtuin‐6 (SIRT6) is a histone‐3 lysine‐9 deacetylase (Michishita et al., 2008) and an epigenetic co‐repressor of HIF (Zhong et al., 2010). In SIRT6 deficient cells, HIF dependent glycolytic gene levels were increased, while mitochondrial respiration was suppressed (Zhong et al., 2010).

N‐terminal isoform of PGC1α (NT‐PGC1α) was shown to have the regulatory role in mitochondrial biogenesis and, thereby promoting adaptation of muscle metabolism induced by endurance exercise (Wen et al., 2014). Thus, detecting NT‐PGC1α levels may show one of mechanisms underlying adaptation induced by exercise under intermittent hypoxia.

Monocarboxylate transporters (MCTs) are transmembrane proteins. Two isoforms, MCT1 and MCT4, transport lactate in and out of cells, respectively, were commonly found in skeletal muscles and the heart (Halestrap & Prince, 1999). Exercise training was shown to enhance MCT1 in human skeletal muscles (Dubouchaud et al., 2000). Chronic hypobaric hypoxia (4300 m for 8 weeks) was shown to enhance and suppress MCT4 protein, respectively, in the heart and plantaris muscle (McClelland & Brooks, 2002). In contrast, chronic hypobaric exposure (5500 m for 3 weeks) was shown to upregulate MCT4 in soleus muscle but not in the heart (Guillaume et al., 2005). However, effects of exercise training under hypoxia or intermittent hypoxia on levels of MCTs has not yet been established.

In the present study, experiments were designed to (1) investigate the effects of acute exercise under short‐duration intermittent hypoxia on muscle mRNA and nuclear protein levels, and (2) clarify how exercise training under intermittent hypoxia has additive effects on improvement of endurance capacity.

## 2 MATERIALS AND METHODS

### 2.1 Animals

Male MCH(ICR)/jcl mice (10 weeks old, 20 and 47 mice for the Experiment‐1 and ‐2, respectively) were purchased from Clea Japan and housed under conditions of a controlled temperature (24 ± 1°C) and relative humidity of approximately 50%. Lighting (7:00–19:00) was controlled automatically. All mice were given commercial laboratory chow (solid CE‐2; Clea Japan) and tap water ad libitum. After mice had been fed for 2 weeks and allowed to adapt to the new environment, they were assigned to each experiment.

### 2.2 Experiment‐1. Acute responses to exercise under intermittent hypoxia

During the second week of the adaptation period, all mice were subjected to treadmill walking using a rodent treadmill (KN‐73; Natsume, Tokyo, Japan) for 5 min per day at 10–15 m min^−1^ with a 5 (π/180) rad incline for 3 days. Mice were randomly assigned to a sedentary control group (Cnt, *n* = 4), acutely exercised group (Ex, run at 18 m min^−1^, *n* = 4), acutely excised under hypoxia group (Hypo, run at 16 m min^−1^ in 14% O_2_, *n* = 4, Figure S1a), acutely exercised under normoxia followed by hypoxia group as shown in Figure S1b (Alt, *n* = 4), or acutely exercised under intermittent hypoxia group as shown in Figure S1c (Int, *n* = 4). The author selected a small sample size in the Experiment‐1 because the effects of exercise under intermittent hypoxia was evaluated for the first time and, therefore, the initial intention was to gather basic evidence regarding the use of this protocol in chronic experimental designs. Endurance exercise lasted for 75 min with a 5 (π/180) rad incline. Instead of using an electrical shock, tail or planta pedis of mice were touched with a conventional test tube blush made by soft procine bristles in order to motivate to run when they stayed on a metal grid more than 2 s. The rodent treadmill described above was covered with a clear plastic film. Mixture of room air and nitrogen gas (100%) was inflated into the chamber to achieve normobaric hypoxia (14% O_2_). The oxygen concentration was monitored using a oxygen sensor (GOX‐100; Greisinger). The air in the chamber was circulated through CO_2_ absorbent (Litholyme; Allied Healthcare Products) to keep CO_2_ concentration in the chamber below 1000 ppm. When O_2_ concentration was raised from 14% to 20.9%, mixture of room air and 100% oxygen gas was inflated into the chamber. The order of each exercise intervention was randomized and mice were randomly assigned to each group. The tissues were collected at 3 h after each treatment. Mice were anesthetized with 3% sevoflurane (193–17791; Fujifilm‐Wako) inhalation. A toe pinch response was used to validate adequate anesthesia. The gastrocnemius muscle was excised and the deep red region (Gr) of the gastrocnemius was isolated from the superficial white region (Gw). Mice were killed by excision of the heart. All tissue samples were frozen in liquid nitrogen and stored at −80°C until later analyses.

### 2.3 RNA isolation and cDNA synthesis

The mRNA‐containing fraction was isolated using RNAzol RT (Molecular Research Center), and 4.5 μg of the fraction was taken for cDNA synthesis using an oligo dT primer and Mmlv reverse transcriptase (RNase H minus point mutant [ReverTra Ace; Toyobo]).

### 2.4 Real‐time PCR analyses

mRNA expression levels were determined by a standard real‐time polymerase chain reaction using the KAPA SYBR FAST qPCR Kit (KAPA Biosystems). Retention in endoplasmic reticulum‐1 (Rer1) was used as endogenous controls for mRNA expression analyses. The PCR conditions were: 1 min pre‐denaturation at 95°C, and then 10 s denaturation at 95°C, 20 s annealing at 60, 60.5 or 63°C, and 1 s extension at 72°C for 40 cycles. A real‐time analysis of PCR amplification was performed on a CFX96 real‐time PCR system and analyzed with the CFX Manager software (Bio‐Rad). Serial 5‐fold dilutions of a cDNA sample were used to generate a standard curve. Non‐specific products such as primer dimer formation were checked by dissociation curves and the results of negative control samples without cDNA. The sequences of the forward and reverse primer sets were as follows: Rer1 (GenBank accession number NM‐026395.1, forward: 5′‐ACCGGAGCTGCGAGTTACAGAA‐3′, reverse: 5′‐ TAGACTTGTCCAGCCAGGACTGA‐3′); VEGFA (NM_001025250.3, forward: 5’‐GCACTGGACCCTGGCTTTACTGCTG‐3′, reverse: 5′‐ACGGCAATAGCTGCGCTGGTAGAC ‐3′); FIH (NM‐176958.3, forward: 5′‐TGCTCATTGGCATGGAAGGAAA‐3′, reverse: 5′‐TGTCACAGGGGTGATGGACA‐3′); SIRT6 (NM‐181586.3, forward: 5′‐TAGAACGCATGGGCTTCCTCA‐3′, reverse: 5′‐AACCGTGTCTCTGACGTACTGC‐3′).

### 2.5 Sample preparation for biochemical analyses

Frozen tissue powder was obtained using a frozen sample crusher (SK mill; Tokken) and homogenized with ice‐cold medium (10 mM HEPES buffer, pH 7.4; 1% NP‐40 [Fujifilm‐Wako]; 11.5% [w/v] sucrose; and 5% [v/v] protease inhibitor cocktail [P2714; Sigma‐Aldrich]) in a ultrasonic bath (43 kHz, 50 W) at 4°C for 5 min, and then gently rotated at 4°C for 10 min. After centrifugation at 18,000*g* and at 4°C for 10 min, the supernatant, cytoplasmic fraction, was collected and stored at −80°C. The pellet was resuspended with ice‐cold buffer described above without NP‐40, and rotated at 4°C for 10 min. After centrifugation as described above, the supernatant was drained, the pellet was resuspended with ice‐cold buffer (10 mM HEPES buffer, pH 7.4; 1.5 mM MgCl_2_, 420 mM NaCl, 25% glycerol and 5% [v/v] protease inhibitor cocktail) and rotated at 4°C for 10 min. After centrifugation as described above, the supernatant, nuclear extraction, was used for western blot analysis. Total protein concentrations were measured using PRO‐MEASURE protein measurement solution (iNtRON Biotechnology).

### 2.6 Western blot analyses

The efficacy of cytoplasmic or nuclear protein separation was confirmed by Western blot (Figure S2) using anti‐GAPDH antibody (a cytoplasmic marker, sc‐166574; Santa Cruz Biotechnology) and anti‐Lamin A/C antibody (a nuclear marker, sc‐376248). The nuclear extraction was used for Western blot analyses. A sample (30 μg of protein) was fractionated by SDS/PAGE on 7.5% or 12% (w/v) polyacrylamide gels (TGX StainFree FastCast gel; Bio‐Rad), exposed to UV for 1 min, and total protein patterns were visualized using ChemiDoc MP Imager (Bio‐Rad). The stain‐free gel contains a trihalo compound which reacts with proteins during separation, rendering them detectable using UV exposure (Gilda & Gomes, 2013). Then, gels were electrophoretically transferred to a polyvinylidene fluoride membrane. The blots were blocked with 5% (w/v) non‐fat dry milk (sc‐2325; Santa Cruz Biotechnology) in 0.1 M phosphate‐buffered saline (PBS) with 0.05% Tween20 for 1 h, and then exposed to a specific primary antibody (Santa Cruz) against NT‐PGC1α (1:1000, sc‐518025), PHD2 (1:1000, sc‐271835), and heat shock factor‐1 (HSF1, 1:1000, sc‐17757) diluted in blocking buffer for 1 h. After the blots had been incubated with a HRP‐labeled mouse IgGκ light chain binding protein (1:5000, sc‐516102, Santa Cruz), they were reacted with Clarity Western ECL substrate (Bio‐Rad), Clarity Max Western ECL substrate (Bio Rad), or their mixture. The required proteins were detected with ChemiDoc MP. The densities of the specific bands were quantified using Image Lab software (Bio Rad) and normalized to the densities of all protein bands in each lane on the membrane (Gilda & Gomes, 2013). Then, the normalized densities of the bands were normalized again to the same sample that was run on every gel and transferred to every membrane (Suzuki, 2021).

### 2.7 Experiment‐2: Chronic response of exercise under intermittent hypoxia

Forty‐seven male mice (10 weeks old) were randomly assigned to the sedentary control group (Sed, *n* = 12) and training group (*n* = 35). In order to familiarize mice with the treadmill device, mice in the training group were subjected to treadmill walking three times a week during the second week of the acclimation period using a controlled treadmill (Modular motor assay, Columbus Instruments, Columbus, OH, USA) for 3 min per day at 10–15 m min^−1^ with a 5 (π/180) incline. Following the acclimation period, mice in the training group were subjected to a maximal exercise capacity test with a graded ramp running protocol using the controlled treadmill, as shown in Figure S3. Total work (J, kg•m^2^•s^−2^) was calculated by the product of body weight (kg), speed (m s^−1^), time (sec), slope (%), and 9.8 (m s^−2^). Exhaustion was defined when the mouse stayed for more than 5 s on the metal grid (no electrical shock) at the rear of the treadmill, despite external gentle touch being applied to their tail with a conventional elastic bamboo stick (0.8 mm in diameter). Following the performance test, mice were given a 48‐h non‐exercise period prior to treadmill training.

**FIGURE 1:**
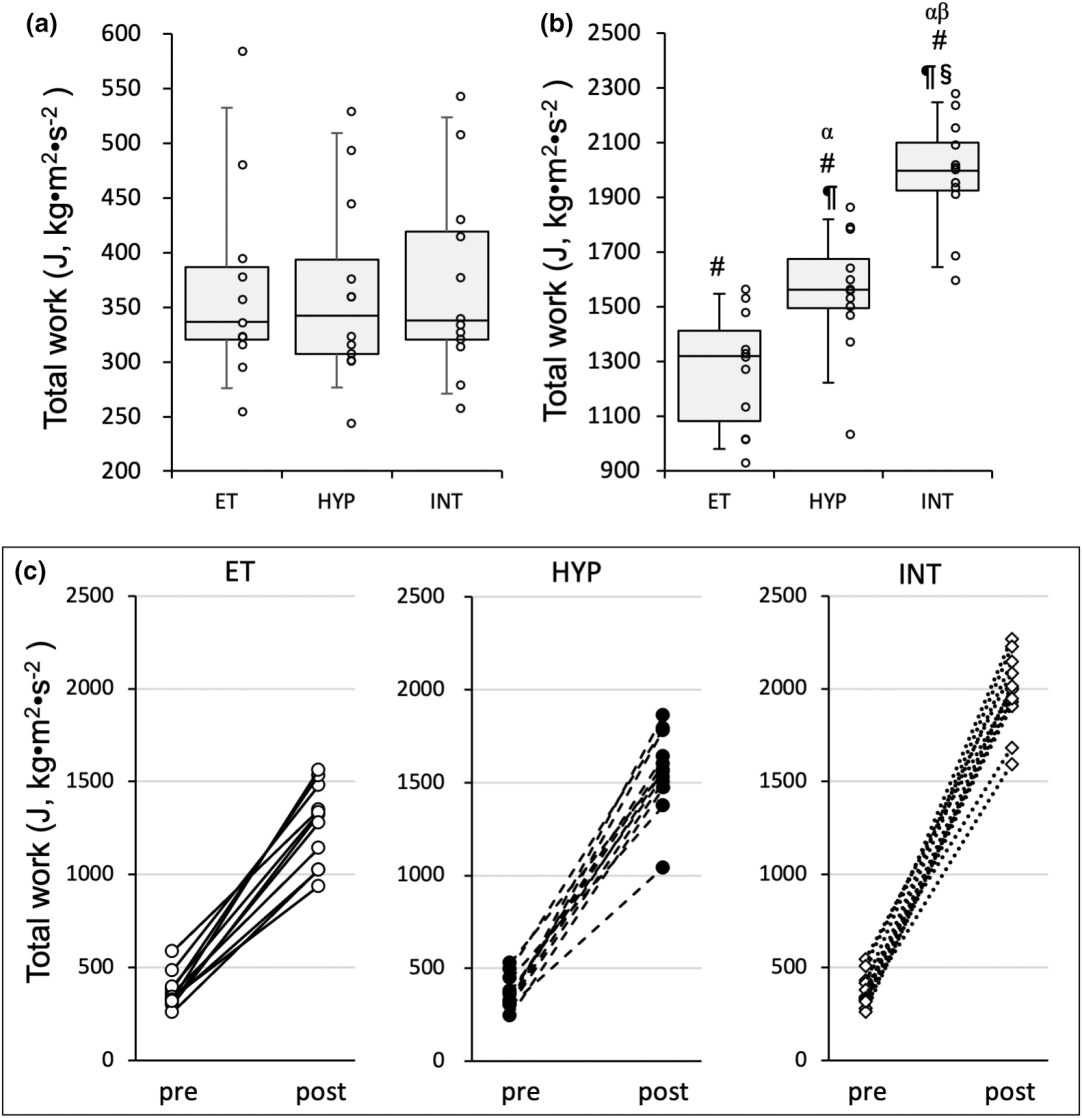
Endurance exercise performance test. Total work capacity of the endurance capacity test (a) before and (b) after 4 weeks of treadmill exercise training. ^#^, Significantly different from pre‐treadmill training values of each group shown in the panel (a). Significantly different from ^¶^ET and ^§^HYP groups. The 95% confidential interval did not contain the mean value of the ^β^ET and ^γ^HYP groups. Values are expressed as box and whisker plots with 5th, 25th, 50th, 75th and 95th percentile. Dots are individual data points. (c) Individual changes in total work values before and after the training

Mice in the training group were divided into a normoxic exercise‐trained group (ET, *n* = 11), hypoxic exercise‐trained group (HYP, *n* = 12), or exercise‐trained under intermittent hypoxia group as shown in the Figure S1c (INT, *n* = 12) in order to match the mean and standard deviation (SD) values of pre‐training results of total work (Figure 1a). All mice in the trained groups underwent successful exercise training, thus they were included in the present results. Mice in the training groups were subjected to endurance exercise training for 4 weeks, 6 days per week. Mice in the HYP and INT groups ran normoxic exercise as same protocols as in the ET group on the 3 days per week (Mon, Wed, and Fri). On the other days (Tue, Thu, and Sat), the HYP and INT groups ran under hypoxia or intermittent hypoxia, respectively. For normoxic exercise, mice ran for 75 min at 18 m min^−1^ with a 10 (π/180) rad incline on the first day of training. The speed was gradually increased by 1 m min^−1^ on every 3 days of the training period. When mice in HYP and INT groups exercised under hypoxia, the run speed was set at the values obtained by subtracting 2 m min^−1^ from the speed of normoxic exercise to set the relative workload equivalently between normoxic and hypoxic exercise regimens. Each exercise intervention was carried out between 5 am to 9 am, and the order of each intervention was randomized daily. Forty‐eight hours after the last training bout, maximal exercise capacity was determined as described above.

Forty‐eight hours after the performance test, mice were anesthetized as described in the Experiment‐1. The soleus (SOL), plantaris (PL), and gastrocnemius muscles were excised and the deep red region (Gr) of the gastrocnemius was isolated from the superficial white region (Gw). The diaphragm (DIA) was excised. Blood samples were collected from the beating heart, and hemoglobin concentrations were measured spectrophotometrically (Hemoglobin‐kit, Wako Pure Chemical Industries). All samples were frozen in liquid nitrogen for biochemical analyses. The remaining muscles, that is, those on the right side, were excised and placed in embedding medium, O.C.T. compound (Miles), and then rapidly frozen in isopentane cooled to its melting point (−160°C) with liquid nitrogen. Mice were killed by excision of the heart. The heart was excised, whole heart and left ventricle (LV) were weighed. The apex half of LV was used for biochemical analyses, and the other half was used for histochemical analyses. All tissue samples were stored at −80°C until later analyses.

### 2.8 Histological analyses

Histochemical examinations of capillary profiles and muscle fiber phenotypes were conducted as previously reported by the author with slight modifications (Suzuki, 2019). Briefly, ten‐micrometer‐thick serial cross‐sections were obtained using a cryotome (CM‐1500; Leica Japan) at −20°C from the mid‐belly portion of calf muscles. These sections were air‐dried, fixed with 100% ethanol at 4°C for 15 min, incubated in 0.1 M phosphate‐buffered saline (PBS) with 0.1% Triton X‐100, and washed in PBS. Sections were then blocked with 10% goat normal serum at room temperature for 30 min, washed in PBS for 5 min, and incubated at 4°C overnight with a mixture of an anti‐type I myosin heavy chain (MHC) antibody (BA‐F8; mouse IgG2b, 1:80), and anti‐type IIA MHC antibody (SC‐71, mouse IgG1, 1:80) diluted with PBS. Sections were then reacted with Alexa Fluor 350‐labeled anti‐mouse IgG2b (1:500), Alexa Fluor 647‐labeled anti‐mouse IgG1 (1:500), and fluorescein‐labeled Griffonia simplicifolia lectin (GSL‐I) (1:100, [FL 1101; Vector Laboratories]) diluted with PBS at room temperature for 2 h. Sections were coverslipped with Fluoromount/Plus (K048; Diagnostic BioSystems). Primary and secondary antibodies were purchased from the Developmental Studies Hybridoma Bank (University of Iowa) and Thermo Fisher Scientific, respectively. Fluorescent images of the incubated sections were observed using a microscope (Axio Observer; Carl Zeiss Japan). Muscle fiber phenotypes were classified as type I (blue), type I + IIA (faint blue and faint red), type IIA (red), type IIAX (faint red), and type IIB + IIX (unstained). Fluorescent images were obtained from SOL, PL, the lateral (GrL) and medial (GrM) portions of Gr, and Gw. The negative control without primary antibodies was confirmed to show no fluorescent signal.

For histochemical examinations of capillary profiles in LV, cross‐sections were obtained as described above. These sections were air‐dried, fixed with 100% ethanol at 4°C for 15 min, incubated in PBS with 0.1% Triton X‐100, and washed in PBS. The sections were incubated with a fluorescein‐labeled GSL‐I in PBS for 2 h at room temperature. To stain plasma membrane, the sections were then incubated with a CF594‐labeled wheat germ agglutinin (29023; Biotium) diluted with Hank's balanced salt solution without glucose and phenol red for 45 min. Sections were coverslipped as described above.

Non‐overlapping microscopic fields were selected at random from each tissue sample. The observer was blinded to the source (groups) of each slide during the measurements using a random number table.

### 2.9 Biochemical analyses of enzyme activity

The activity of 3‐hydroxyacyl‐CoA‐ dehydrogenase (HAD) was assayed according to the method of Bass et al. (1969). The activity of citrate synthase (CS) was assayed according to the methods of Srere (1969). Pyruvate dehydrogenase complex (PDHc) activity was assayed according to the method of Ke et al. (2014). The activity of carnitine palmitoyl transferase (CPT)‐2 was assayed as previously reported (Suzuki, 2021). Specific lactate dehydrogenase activities, pyruvate‐to‐lactate (LDH‐PL) or lactate‐to‐pyruvate (LDH‐LP) conversions, were determined according to Howell et al. (1979) with following modifications. LDH‐PL reaction was done in the presence of 240 mM sodium pyruvate and 0.1 mM NADH in 50 mM Triethanolamine‐HCl buffer (pH 7.6). LDH‐LP reaction was performed in the presence of 70 mM lactic acid and 1.4 mM NAD+ in Tris–HCl buffer (pH 7.6). All measurements were conducted at 25°C with a spectrophotometer (U‐2001; Hitachi Co.), and enzyme activities were obtained as micromoles hour^−1^ milligram of protein^−1^. Total protein concentrations were measured as described above.

### 2.10 Western blot analyses

Nuclear levels of NT‐PGC1α, PHD2, and HSF1 proteins were determined as mentioned in the Experiment‐1. Using cytoplasmic fraction of protein (60 μg), protein levels of fatty acid binding protein (FABP) (1:1000, sc‐514208), MCT1 (1:1000, sc‐365501), and MCT4 (1:1000, sc‐376140) was determined.

### 2.11 Statistical analyses

**FIGURE 2:**
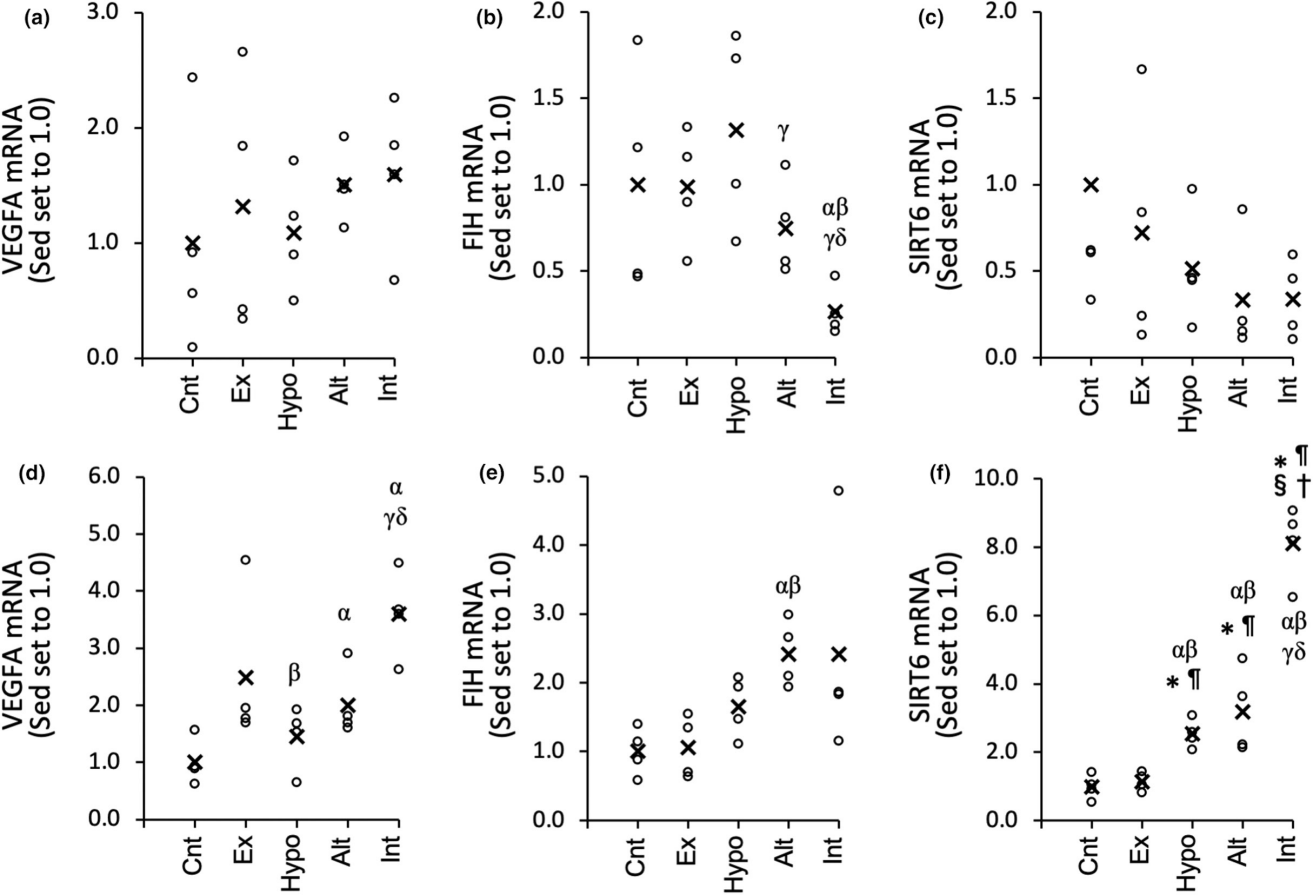
mRNA expression levels of VEGFA (a, d), FIH (b, e), and SIRT6 (c, f) in the red (a, b, c) and white (d, e, f) regions of the gastrocnemius muscle. a. u., arbitrary unit. Values are represented as individual data plot (circle) and mean value (x). The number of mice was 4 per group. Significantly different from the *Cnt, ^¶^Ex, ^§^Hypo, and ^†^Alt groups. The 95% confidential interval did not contain the mean value of the ^α^Sed, ^β^ET, and ^γ^HYP groups

**FIGURE 3:**
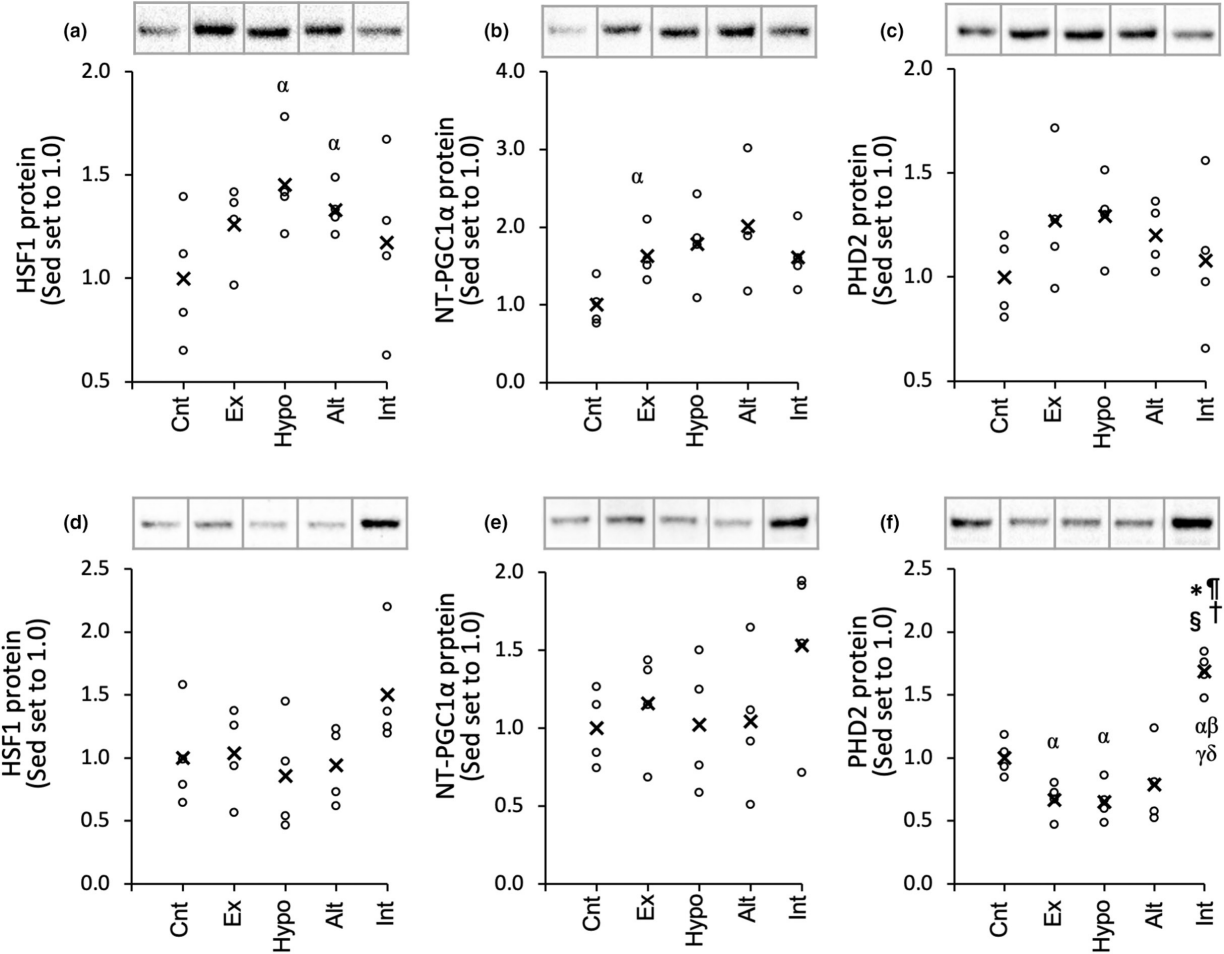
Protein levels for HSF1 (a, d), NT‐PGC1α (b, e), and PHD2 (c, f) in the red (a, b, c) and white (d, e, f) regions of the gastrocnemius muscle. a. u., arbitrary unit. Values are represented as individual data plot (circle) and mean value (x). The number of mice was 4 per group. Significantly different from the *Cnt, ^¶^Ex, ^§^Hypo, and ^†^Alt groups. The 95% confidential interval did not contain the mean value of the ^α^Sed, ^β^ET, and ^γ^HYP groups

**FIGURE 4:**
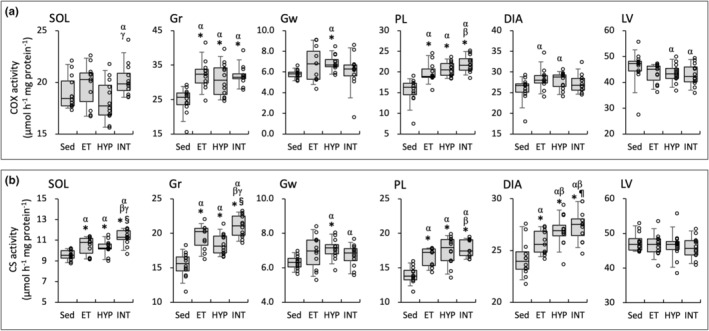
Enzyme activity values for COX (a) and CS (b). Values are expressed as box and whisker plots with 5th, 25th, 50th, 75th and 95th percentile. Dots are individual data points. Significantly different from *Sed, ^¶^ET, and ^§^HYP groups. The 95% confidential interval did not contain the mean value of the ^α^Sed, ^β^ET, and ^γ^HYP groups

**FIGURE 5:**
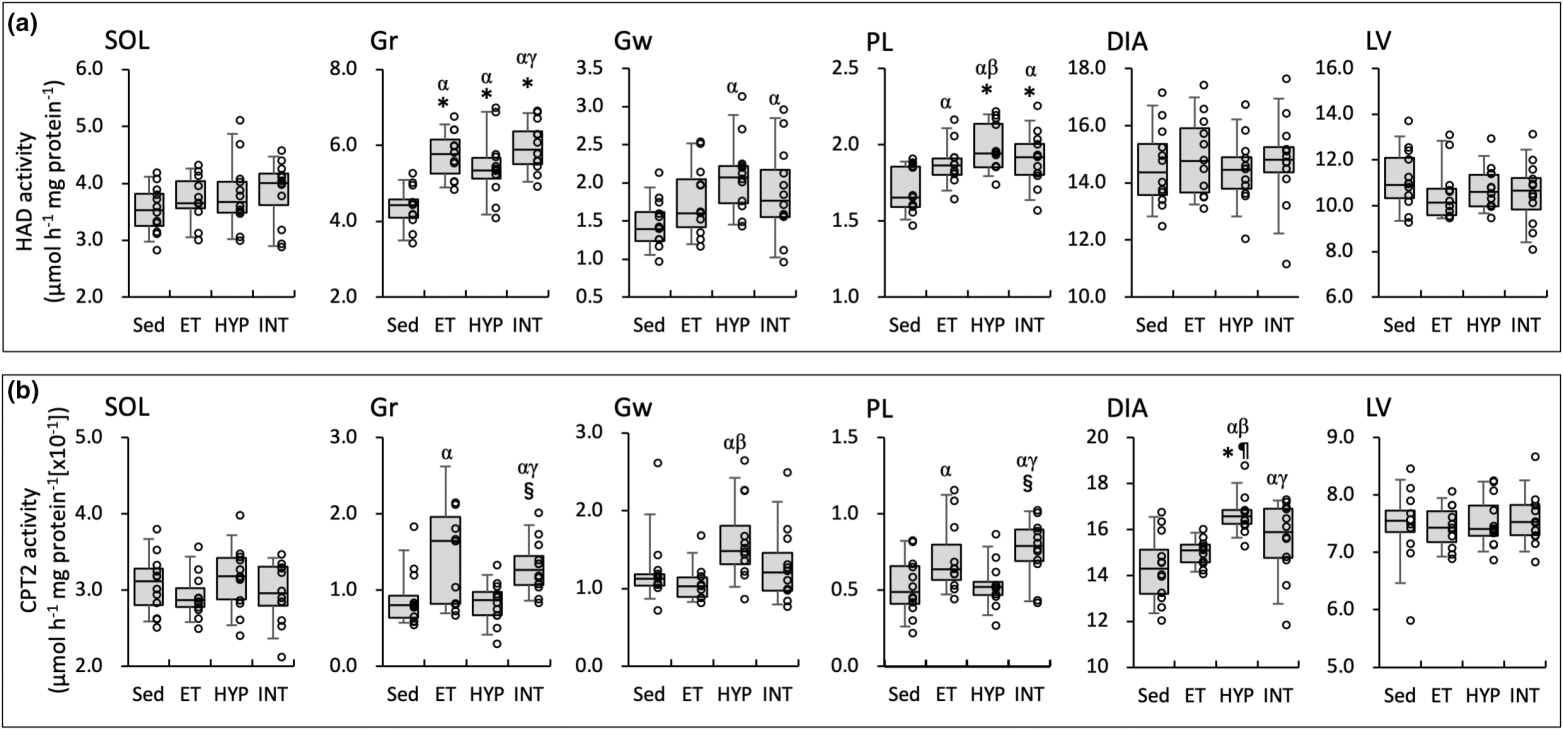
Enzyme activity values for HAD (a) and CPT2 (b). Values are expressed as box and whisker plots with 5th, 25th, 50th, 75th and 95th percentile. Dots are individual data points. Significantly different from *Sed, ^¶^ET, and ^§^HYP groups. The 95% confidential interval did not contain the mean value of the ^α^Sed, ^β^ET, and ^γ^HYP groups

**FIGURE 6:**
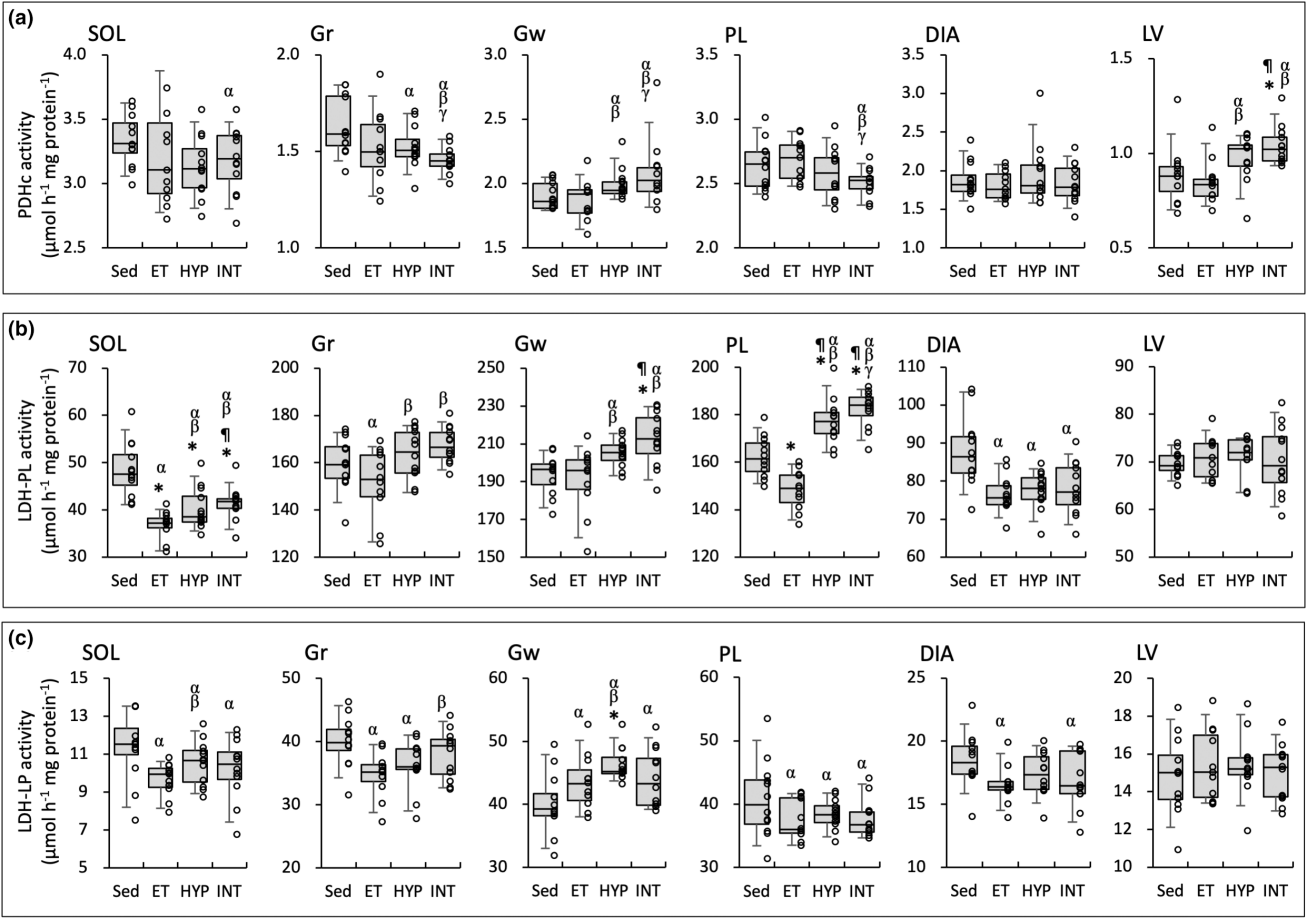
Enzyme activity values for PDHc (a), LDH‐PL (b), and LDH‐LP (c). Values are expressed as box and whisker plots with 5th, 25th, 50th, 75th and 95th percentile. Dots are individual data points. Significantly different from *Sed and ^¶^ET groups. The 95% confidential interval did not contain the mean value of the ^α^Sed, ^β^ET, and ^γ^HYP groups

**FIGURE 7:**
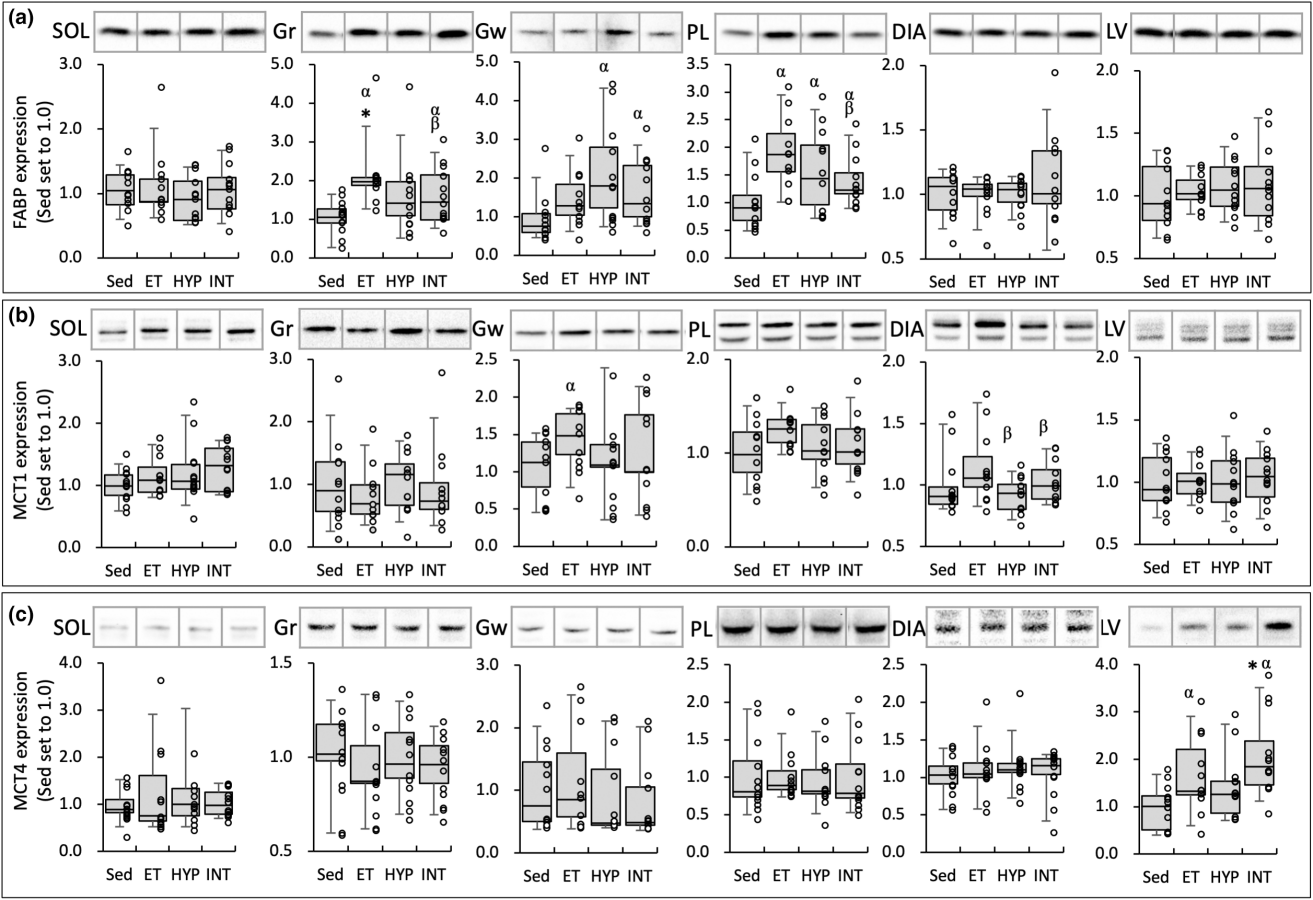
Protein levels for FABP (a), MCT1 (b), and MCT4 (c). a. u., arbitrary unit. Values are expressed as box and whisker plots with 5th, 25th, 50th, 75th, and 95th percentile. Dots are individual data points. Significantly different from *Sed group. The 95% confidential interval did not contain the mean value of the ^α^Sed group

**FIGURE 8:**
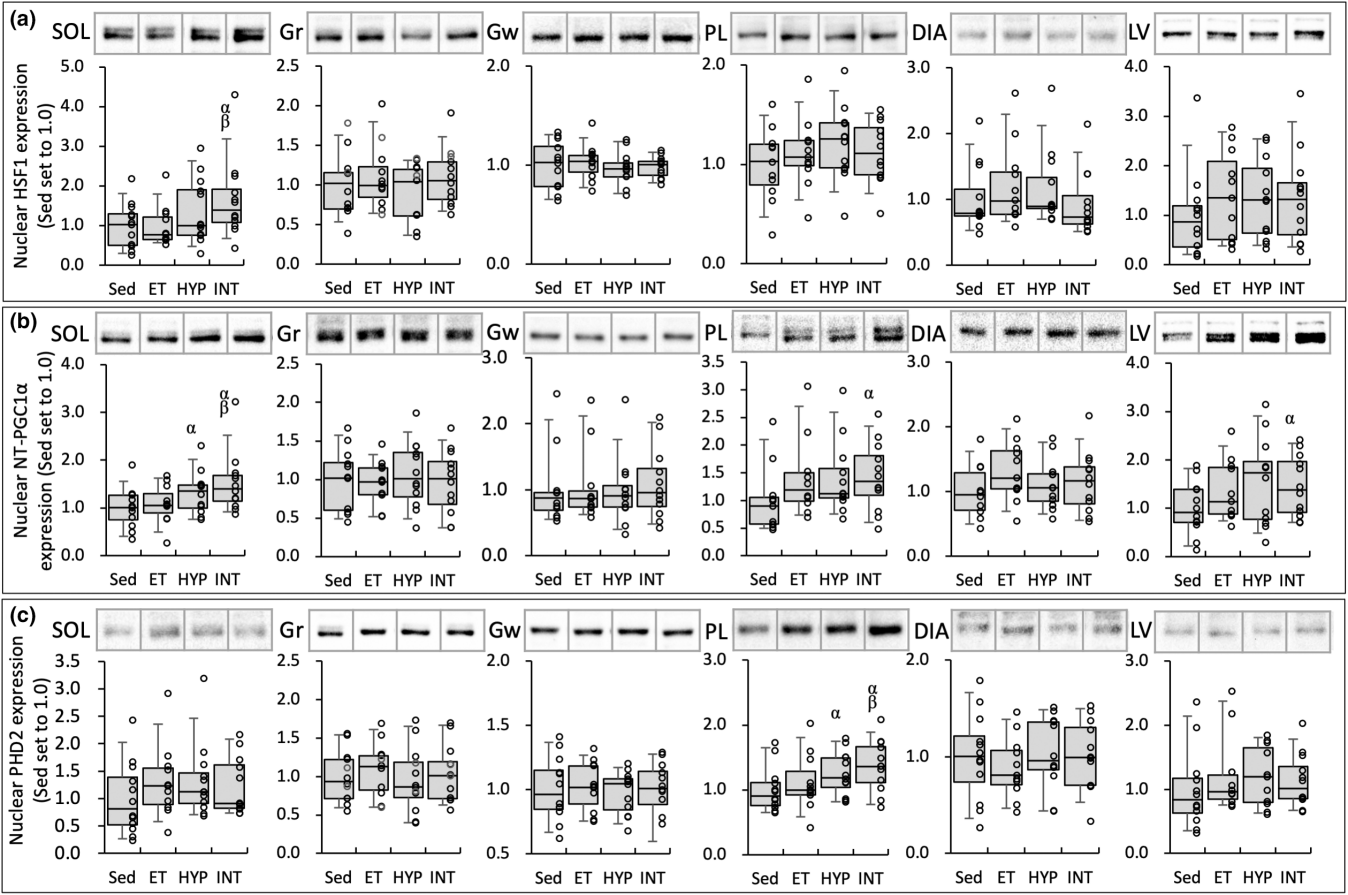
Nuclear protein levels for HSF1 (a), NT‐PGC1α (b), and PHD2 (c). a. u., arbitrary unit. Values are expressed as box and whisker plots with 5th, 25th, 50th, 75th, and 95th percentile. Dots are individual data points. The 95% confidential interval did not contain the mean value of the ^α^Sed and ^β^ET groups

According to the statement of American Statistical Association (Wasserstein & Lazar, 2016), the present study used the Bayesian data analysis for statistical analysis, instead of *p* value as in null hypothesis significance testing, such as ANOVA and post‐hoc test. Differences between the two groups were examined using Bayesian estimation with a gamma prior distribution proposed by Kruschke (2015). The posterior distribution was obtained using the Markov chain Monte Carlo (MCMC) methods. Public domain R, RStudio, and JAGS programs were used for computing Bayesian inference. The MCMC chains were considered to show a stationary distribution when the Gelman‐Rubin values (shrink factor) were less than 1.10 for all parameters. The significance of differences was evaluated by the 95% highest density interval (HDI) and a region of practical equivalence (ROPE) (Kruschke, 2018). When the HDI value on the effect size fell outside of the ROPE set at −0.1 to 0.1, the difference was regarded as significant (Kruschke, 2018; Kruschke & Liddell, 2018). When 95% confidence interval (CI) values did not contain the mean value of target group for comparison, differences were considered to be biologically important (Du Prel et al., 2009). The Pearson's product moment correlation coefficients (*r*) were used to establish correlations using the EZR public domain software (Kanda, 2013). The effect sizes of r value were classified as low (*r* = 0.3), moderate (*r* = 0.5), or high (*r* = 0.7). Data are expressed as individual plots in Figures 2 and 3, both individual plots and box and whisker plots with 5th, 25th, 50th, 75th and 95th percentile in Figures 1 and 4, 5, 6, 7, 8. In Tables, data are expressed as means ± standard deviation (SD) with range in brackets.

## 3 RESULTS

### 3.1 Experiment‐1. Acute response to exercise under hypoxia or intermittent hypoxia

At 3 h after the exercise with or without hypoxic interventions, VEGFA mRNA expression levels in Gw were substantially higher in the Alt (2.0‐fold, CI: 1.04–2.97) and Int (3.6‐fold, CI: 2.38–4.81) groups than in the Cnt group (Figure 2d). The expression of FIH mRNA in Gw was considerably higher in the Alt group (2.4‐fold, CI: 1.63–3.20) than in the Cnt group (Figure 2e). SIRT6 mRNA expression levels in GW were significantly higher in the Int group than in the other four groups (from 2.5‐ to 8.1‐fold, Figure 2f). Moreover, SIRT6 mRNA levels were significantly greater in the Hypo and Alt groups than in the Cnt and Ex groups.

In Gr, VEGFA mRNA levels did not show any substantial change (Figure 2a). FIH mRNA levels were substantially lower in the Int group (from 0.27‐ to 0.36‐fold) than in the other four groups (Figure 2b). SIRT6 mRNA expression levels were lower, but not significantly so, in the Hypo (from 0.51‐ to 0.71‐fold), Alt (from 0.33‐ to 0.65‐fold), and Int (from 0.34‐ to 0.68‐fold) groups than in the Cnt and Ex groups (Figure 2c). Thus, at 3 h after exercise under intermittent hypoxia, considerable increase in VEGF mRNA and drastic increase in SIRT6 mRNA expression levels were observed in glycolytic muscle portion, while substantial decrease in FIH mRNA levels were observed in oxidative muscle portion. Nuclear PDH2 protein levels in Gw were significantly greater in the Int group (from 1.7 to 2.6‐fold) than in the other four groups (Figure 3f).

### 3.2 Experiment 2: Chronic response of exercise training under intermittent hypoxia

#### 3.2.1 Body and organ masses and maximal exercise capacity

The body weights were not significantly different among the groups (Table 1). The absolute and relative weights of whole heart and LV were significantly higher in the three trained groups than in the Sed group. The relative weight of SOL was significantly greater in the INT group than in the Sed group. Hemoglobin (Hb) concentration values were substantially greater in the HYP group than in the Sed and ET groups.

**TABLE 1:** Body and organ masses

	Sed (*n* = 12)	ET (*n* = 11)	HYP (*n* = 12)	INT (*n* = 12)
Body mass (g)				
Pre‐ET	38.8 ± 1.2	37.0 ± 1.7	36.4 ± 1.1	36.5 ± 1.3
	(33.6–38.0)	(34.3–40.4)	(34.5–37.8)	(33.8–38.2)
Post‐ET	38.0 ± 1.4	36.9 ± 1.6	37.0 ± 1.2	36.6 ± 1.9
	(36.0–41.8)	(34.4–40.2)	(34.7–38.8)	(32.2–39.2)
Organ mass (mg)				
Gastrocnemius	168.0 ± 12.8	167.3 ± 8.2	172.5 ± 8.6	165.7 ± 8.3^γ^
	(145.4–190.6)	(156.3–183.2)	(160.2–186.5)	(154.7–183.5)
Plantaris	20.7 ± 1.5	21.1 ± 2.1	22.0 ± 1.7^a^	21.7 ± 2.5
	(18.4–23.9)	(19.2–25.2)	(19.4–23.9)	(18.4–27.5)
Soleus	8.5 ± 0.86	8.5 ± 0.76	8.8 ± 0.63	9.3 ± 0.97^αβ^
	(6.8–10.2)	(6.8–9.5)	(8.1–10.0)	(7.7–11.2)
Whole heart	142.8 ± 8.1	157.2 ± 10.0*^α^	159.5 ± 9.0*^α^	158.0 ± 13.2*^α^
	(123.1–151.6)	(138.9–171.4)	(142.6–172.5)	(132.7–179.4)
Left ventricle	111.5 ± 6.17	121.4 ± 7.2*^α^	124.4 ± 7.3*^α^	123.5 ± 12.6*^α^
	(96.9–119.2)	(109.7–132.0)	(113.5–135.5)	(104.2–145.7)
Organ mass‐to‐body mass ratio (mg g^−1^)				
Gastrocnemius	4.42 ± 0.18	4.54 ± 0.15^α^	4.66 ± 0.19^αβ^	4.57 ± 0.35
	(3.87–4.85)	(4.26–4.81)	(4.40–4.98)	(4.08–5.31)
Plantaris	0.54 ± 0.04	0.57 ± 0.06	0.59 ± 0.04	0.60 ± 0.07^α^
	(0.49–0.61)	(0.51–0.68)	(0.55–0.67)	(0.50–0.77)
Soleus	0.22 ± 0.02	0.23 ± 0.02	0.24 ± 0.02	0.25 ± 0.02*^αβγ^
	(0.18–0.27)	(0.19–0.26)	(0.21–0.28)	(0.22–0.29)
Whole heart	3.76 ± 0.23	4.26 ± 0.19*^α^	4.31 ± 0.22*	4.32 ± 0.29*^αβ^
	(3.34–4.07)	(3.88–4.56)	(4.02–4.66)	(3.98–5.04)
Left ventricle	2.94 ± 0.18	3.29 ± 0.15*^α^	3.36 ± 0.18*	3.38 ± 0.30*^αβ^
	(2.63–3.20)	(3.06–3.48)	(3.07–3.64)	(2.96–4.09)
Hemoglobin (g 100 ml^−1^)	15.2 ± 0.84	15.9 ± 1.9	17.3 ± 1.4^αβ^	16.4 ± 1.9
	(14.0–16.1)	(13.9–18.6)	(14.5–18.9)	(13.8–18.8)

*Note*: Values are presented as means ± SD with range in brackets. Significantly different from the *Sed group using Bayesian data analysis. Pre‐HT and Post‐HT, at the beginning and end, respectively, of the 4 weeks of exercise training. Τhe 95% confidential interval did not contain the mean value of the ^α^Sed, ^β^ET, and ^γ^HYP groups.

Total work values were significantly increased after 4 weeks of treadmill training in three trained groups (Figure 1b). Total work values were significantly greater in the HYP and INT groups than in the ET group. Moreover, the values in the INT group were significantly greater than those in the HYP group. Thus, exercise under short‐duration intermittent hypoxia had additive effects on exercise‐induced improvements in endurance exercise capacity. Hb values were not correlated with total work values at the end of the experiment (*r* = 0.19, Table 2).

**TABLE 2:** Correlations with total work values

	Explanatory variable	*r*	*p* value
	Hb	0.190	0.34
SOL	CS	0.440	<0.001
	LDHPL	0.558	<0.001
	LDHLP	0.357	0.036
Gw	LDHPL	0.419	0.012
Gr	CS	0.346	0.042
	LDHPL	0.352	0.038
	LDHLP	0.317	0.064
PL	PDHc	−0.524	<0.001
	LDHPL	0.673	<0.001
	FABP	0.414	0.013
DIA	CS	0.607	<0.001
LV	PDHc	0.419	0.012
	C:F ratio	0.432	<0.001

*Note*: r, Pearson's product moment correlation coefficient.

### 3.3 Enzyme activity

COX activity values were significantly higher in the three trained groups than in the Sed group in Gr and PL (Figure 4a). In Gw, COX levels were significantly greater in the HYP group than in the Sed group. COX levels in SOL were substantially higher in the INT group than in the Sed (CI: 1.01–1.12) and HYP (CI: 1.05–1.17) groups. In SOL, Gr, PL, and DIA, CS activity values were significantly higher in the three trained groups than in the Sed group (Figure 4b). In SOL and Gr, CS values showed significantly higher values in the INT group than in the HYP group. Moreover, in these muscles, CS levels were substantially higher in the INT group (SOL, CI: 1.03–1.12; Gr, CI: 1.03–1.13) than in the ET group. In SOL, Gr, and DIA, CS values were positively correlated with total work values (Table 2).

HAD activity values in Gr were significantly higher in the three trained groups than in the Sed group. In PL, the values were significantly higher in the HYP and INT groups than in the Sed group (Figure 5a). In Gr and PL, CPT2 activity levels were significantly higher in the INT group than in the HYP group (Figure 5b). CPT2 values in the HYP group were significantly greater in DIA, and were substantially higher in Gw (CI: 1.05–1.59 and 1.20–1.81, respectively), than those in the Sed and ET groups.

In LV, PDHc activity values were significantly higher in the INT group than in the Sed and ET groups (Figure 6a). Moreover, the values were positively correlated with total work values (Table 2). PDHc levels were substantially lower in the INT group than in the other three groups in Gr (CI: 0.87–0.92 vs Sed; 0.93–0.98 vs ET; 0.93–0.98 vs HYP) and PL (CI: 0.93–0.98 vs Sed; 0.91–0.96 vs ET; 0.95–0.99 vs HYP). PDHc values in PL were negatively correlated with total work values (Table 2). In Gw, LDH‐PL activity values were significantly greater in the INT group than in the Sed and ET groups (Figure 6b) and were positively correlated with total work values (Table 2). In SOL, LDH‐PL levels were significantly lower in the three trained groups than in the Sed group (Figure 6b), but the values were significantly higher in the INT groups than in the ET group. Both LDH‐PL and LDH‐LP levels in SOL and Gr were positively correlated with total work values (Table 2). LDH‐PL activity values in PL were significantly higher in the HYP and INT groups than in the Sed and ET groups, and were positively correlated with total work values (Table 2). In HYP group, LDH‐LP activity values in Gw were significantly greater (by 15%) than those in the Sed group (Figure 6c).

### 3.4 Protein levels

MCT4 protein levels in LV were significantly greater in the INT group (2.1‐fold) and were considerably higher in the ET group (1.6‐fold) than in the Sed group (Figure 7c). In Gw, MCT1 levels were substantially higher in the ET group (1.4‐fold; CI: 1.10–1.67) than in the Sed group (Figure 7b). FABP levels in Gr were significantly greater in the ET (2.1‐fold) group and were substantially greater in the INT groups (1.6‐fold, CI: 1.12–2.08) than in the Sed group (Figure 7a). In Gw, FABP levels were substantially higher in the HYP (2.3‐fold, CI: 1.48–3.21) and INT (1.8‐fold, CI: 1.24–2.38) groups than in the Sed group. In PL, FABP levels were substantially higher in the ET (1.9‐fold, CI: 1.42–2.34), HYP (1.5‐fold, CI: 1.02–2.01), and INT (1.4‐fold, CI: 1.07–1.22) groups than in the Sed group and were positively correlated with total work values (Table 2). Nuclear NT‐PGC1α levels in SOL were considerably greater in the HYP (1.3‐fold, CI: 1.04–1.53) and INT (1.5‐fold, CI: 1.09–1.79) groups than in the Sed group (Figure 8b). Moreover, the levels were substantially greater in the INT group (1.4‐fold, CI: 1.006–1.66) than in the ET group. NT‐PGC1α levels were considerably greater in the INT group than in the Sed group in PL (1.4‐fold, CI: 1.04–1.82) and LV (1.5‐fold, CI: 1.06–1.85). Levels of nuclear HSF1 protein in SOL were substantially greater in the INT group than in the Sed (1.6‐fold, CI: 1.004–2.27) and ET (1.7‐fold, CI: 1.02–2.31) groups (Figure 8a). Nuclear PHD2 levels in PL were considerably greater in the HYP (1.2‐fold, CI: 1.03–1.46) and INT (1.4‐fold, CI: 1.11–1.62) groups than in the Sed group (Figure 8b). Moreover, the levels were substantially greater in the INT group (1.2‐fold, CI: 1.03–1.46) than in the ET group.

### 3.5 Muscle fiber‐type composition

**FIGURE 9:**
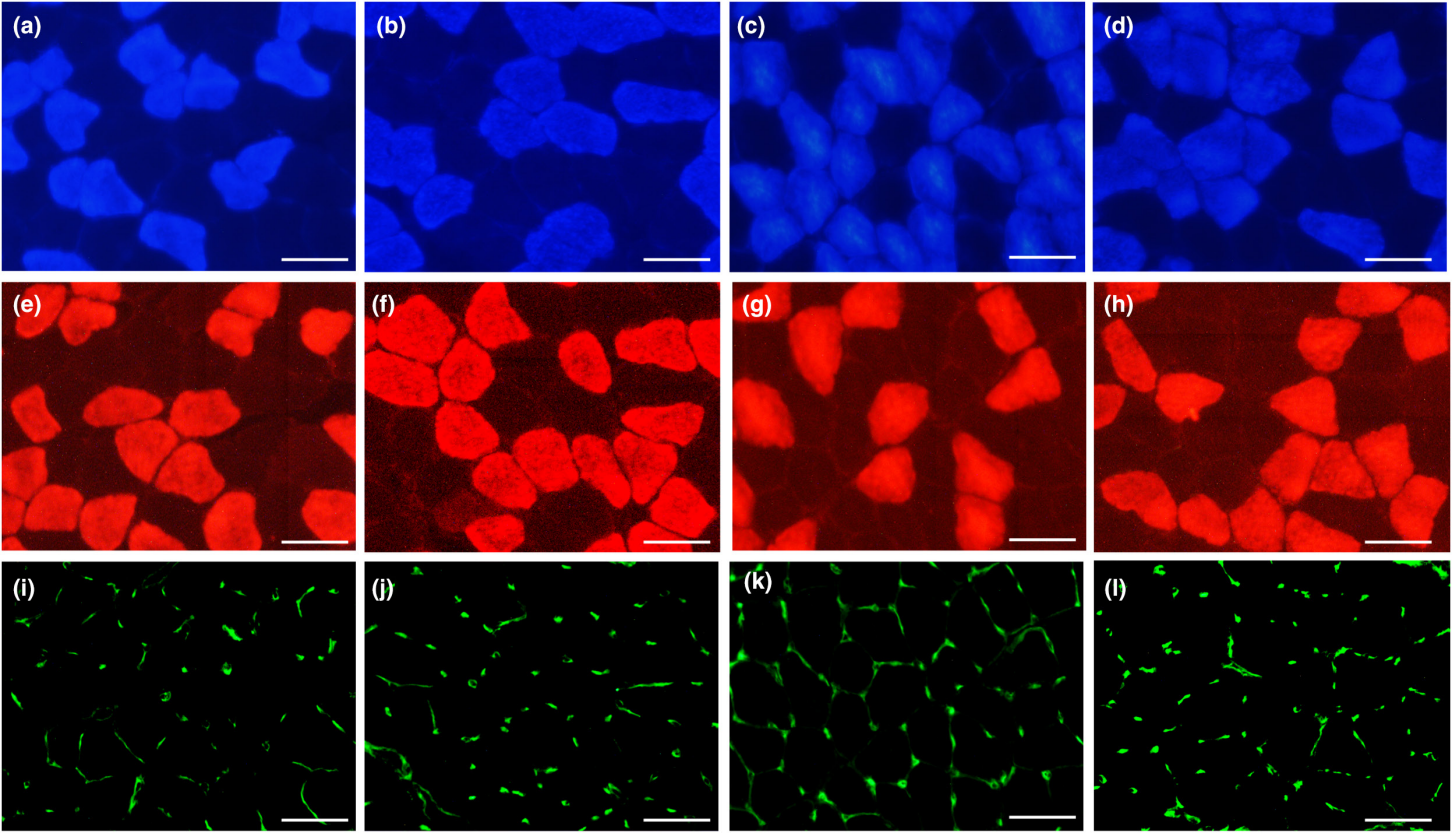
Representative immunofluorescent images of SOL muscle. (a–d) anti‐type I MHC antibody; (e–h) anti‐type IIA MHC antibody; (i–l) capillary profile; (a, e, i) Sed, (b, f, j) ET, (c, g, k) HYP, and (d, h, l) INT groups. Horizontal bars represent 50 μm

Representative immunofluorescent images for muscle fiber and capillary profiles in SOL were shown in Figure 9. The proportion of type I fibers in SOL (CI: 1.04–1.19) and IIAX fibers in GrL (CI: 1.29–3.09) was substantially higher in the HYP group than in the Sed group (Table 3). Thus, exercise under hypoxia considerably increased the proportion of type I fibers in slow‐twitch muscle.

**TABLE 3:** Fiber type composition values (%)

	Type	Sed (*n* = 12)	ET (*n* = 11)	HYP (*n* = 12)	INT (*n* = 12)
SOL	I	49.3 ± 7.2	52.5 ± 8.1	55.1 ± 6.0^α^	51.5 ± 8.4
		(40.4–62.2)	(42.3–67.6)	(41.9–61.4)	(38.0–68.3)
	IIA	42.9 ± 6.5	41.4 ± 6.4	40.7 ± 4.0	42.9 ± 6.0
		(32.0–53.0)	(30.3–49.8)	(34.6–48.9)	(30.1–52.8)
	I + IIA	0.44 ± 0.49	0.35 ± 0.48	0.29 ± 0.31	0.57 ± 0.78
		(0.0–1.3)	(0.0–1.6)	(0.0–0.9)	(0.0–2.7)
	IIAX	4.0 ± 2.7	4.2 ± 2.4	3.0 ± 2.6	3.5 ± 3.3
		(0.9–9.0)	(0.8–9.3)	(0.0–8.3)	(0.0–12.2)
	IIB + IIX	3.3 ± 3.2	1.8 ± 1.9	1.0 ± 1.1^αβ^	1.5 ± 1.6 ^α^
		(0.0–10.4)	(0.0–5.7)	(0.0–2.9)	(0.0–5.9)
PL	I	4.52 ± 2.2	2.31 ± 2.6^α^	3.69 ± 2.8	3.5 ± 3.1
		(0.3–8.4)	(0.0–6.7)	(0.0–8.7)	(0.6–9.7)
	IIA	44.8 ± 6.7	42.2 ± 8.8	43.1 ± 8.5	44.8 ± 4.9
		(33.3–56.6)	(29.4–54.1)	(32.1–56.7)	(34.6–52.1)
	I + IIA	0.19 ± 0.30	0.17 ± 0.40	0.53 ± 0.80	0.26 ± 0.46
		(0.0–0.8)	(0.0–1.2)	(0.0–2.8)	(0.0–1.6)
	IIAX	4.8 ± 3.3	7.0 ± 3.0^α^	6.3 ± 2.3^α^	7.5 ± 3.4^α^
		(2.4–14.6)	(1.6–11.7)	(2.9–11.1)	(3.3–15.9)
	IIB + IIX	45.7 ± 8.0	48.3 ± 9.8	46.3 ± 8.5	43.9 ± 3.6
		(33.6–58.8)	(33.6–65.9)	(33.3–59.9)	(37.2–50.6)
GrL	I	18.0 ± 2.5	16.9 ± 3.1	19.0 ± 3.0^β^	16.2 ± 3.2^γ^
		(14.4–22.2)	(13.1–23.4)	(13.5–22.6)	(11.8–20.9)
	IIA	42.9 ± 4.2	48.4 ± 3.2^α^	44.8 ± 4.4	48.4 ± 2.6^αγ^
		(36.7–52.0)	(41.8–51.7)	(35.9–51.4)	(42.6–51.4)
	I + IIA	0.10 ± 0.30	0.11 ± 0.37	0.05 ± 0.16	0.08 ± 0.21
		(0.0–0.8)	(0.0–1.2)	(0.0–0.6)	(0.0–0.7)
	IIAX	4.0 ± 5.4	4.6 ± 2.7	8.8 ± 5.7^αβ^	5.6 ± 2.1^αγ^
		(0.0–18.8)	(0.9–10.9)	(1.4–20.3)	(2.6–9.8)
	IIB + IIX	35.0 ± 7.5	30.0 ± 4.4	27.3 ± 8.9	29.7 ± 3.8^a^
		(19.5–44.7)	(23.9–40.9)	(8.3–40.8)	(24.7–36.9)
GrM	I	41.9 ± 7.42	40.6 ± 8.5	39.9 ± 5.8	42.3 ± 9.6
		(28.8–52.5)	(29.9–55.9)	(31.8–50.8)	(26.0–56.4)
	IIA	35.7 ± 5.4	38.9 ± 5.9	43.0 ± 9.6	41.8 ± 6.0
		(28.4–46.0)	(31.2–48.8)	(22.4–55.4)	(34.0–54.3)
	I + IIA	0.32 ± 0.45	0.34 ± 0.44	0.053 ± 0.18	0.69 ± 1.9
		(0.0–1.1)	(0.0–1.4)	(0.0–0.6)	(0.0–6.6)
	IIAX	5.8 ± 5.5	3.3 ± 4.1	3.6 ± 2.8	5.2 ± 4.1
		(0.8–18.9)	(0.0–11.0)	(0.0–8.2)	(0.0–14.0)
	IIB + IIX	16.2 ± 2.4	16.8 ± 8.5	13.4 ± 6.4	10.1 ± 6.8
		(13.7–21.7)	(1.7–28.0)	(1.5–21.9)	(2.2–21.3)
Gw	IIB + IIX	100	100	100	100

*Note*: Values are presented as means ± SD with range in brackets. Significantly different from the *Sed group using Bayesian data analysis. Τhe 95% confidential interval did not contain the mean value of the ^α^Sed, ^β^ET, and ^γ^HYP groups.

### 3.6 Capillarization

**FIGURE 10:**
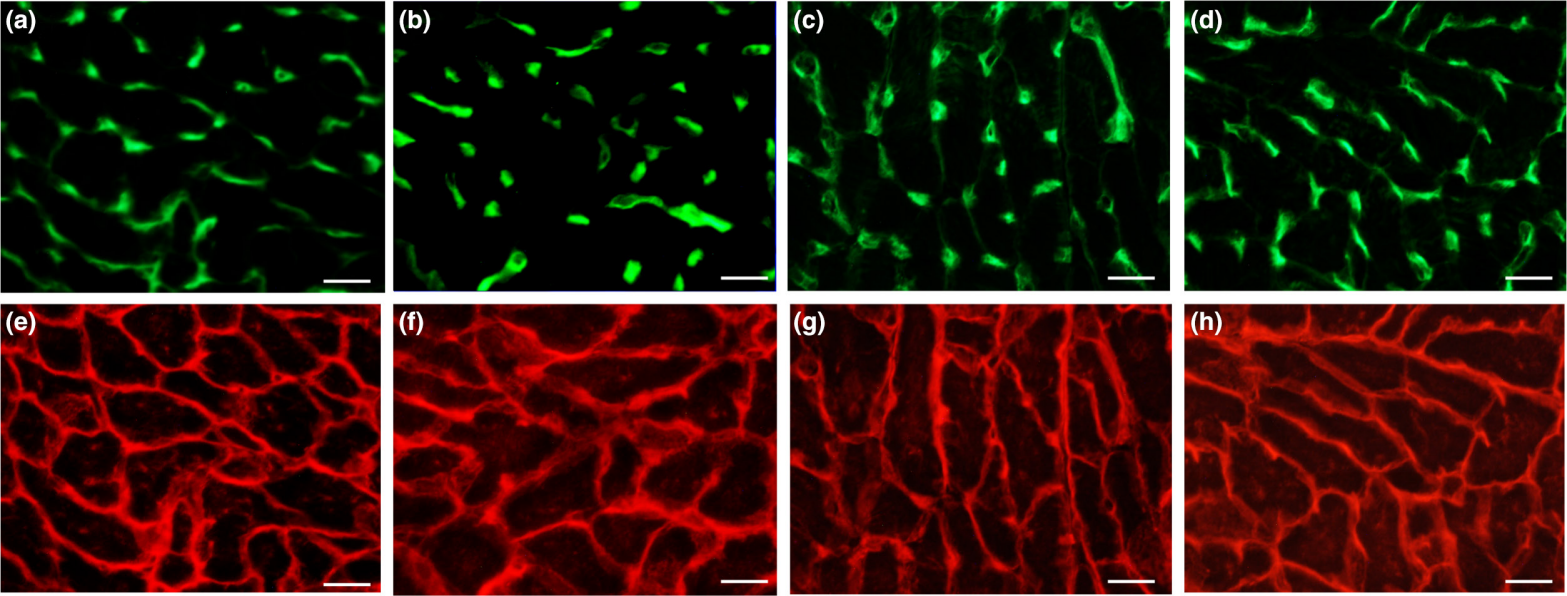
Representative immunofluorescent images of LV. (a–d) capillary profile; (e–h) plasma membrane profile; (a, e) Sed, (b, f) ET, (c, g) HYP, and (d, h) INT groups. Horizontal bars represent 30 μm

Representative immunofluorescent images for capillary and cardiac myocyte in LV were shown in Figure 10. Capillary‐to‐fiber ratio (C:F) values in SOL were significantly higher in the INT group than in the Sed group, and were substantially higher than in the ET (CI: 1.002–1.07) and HYP (CI: 1.05–1.12) groups (Table 4). C:F values in PL were substantially higher in the INT group than in the Sed (CI: 1.02–1.10), ET (CI: 1.02–1.10), and HYP (CI: 1.04–1.11) groups. C:F values in LV were substantially higher in the INT group than in the Sed (CI: 1.10–1.22), ET (CI: 1.04–1.16), and HYP (CI: 1.08–1.20) groups. In LV, C:F values were positively correlated with total work values (Table 2). Capillary density values in SOL, GrL, and GrM were substantially higher in the INT group than in the Sed (CI: 1.06–1.24, 1.07–1.28, 1.008–1.21, respectively) and HYP (CI: 1.06–1.24, 1.01–1.21, 1.007–1.21, respectively) groups. Thus, exercise under short‐duration intermittent hypoxia facilitate exercise‐induced capillary growth in left ventricle and hindlimb muscles.

**TABLE 4:** Capillary profile values

	Sed (*n* = 12)	ET (*n* = 11)	HYP (*n* = 12)	INT (*n* = 12)
Capillary‐to‐fiber ratio				
SOL	1.67 ± 0.12	1.84 ± 0.15^α^	1.76 ± 0.19	1.90 ± 0.09*^αβγ^
	(1.46–1.86)	(1.65–2.13)	(1.57–2.12)	(1.69–2.03)
PL	1.66 ± 0.09	1.67 ± 0.18	1.65 ± 0.14	1.77 ± 0.10^αβγ^
	(1.44–1.79)	(1.43–1.99)	(1.41–1.86)	(1.59–1.95)
GrL	1.93 ± 0.13	2.00 ± 0.16^α^	2.06 ± 0.24	2.15 ± 0.31^α^
	(1.71–2.10)	(1.80–2.28)	(1.75–2.49)	(1.83–2.76)
GrM	2.01 ± 0.23	1.90 ± 0.17	1.85 ± 0.20^α^	2.05 ± 0.33
	(1.74–2.45)	(1.58–2.14)	(1.51–2.20)	(1.61–2.84)
Gw	0.97 ± 0.13	0.99 ± 0.09	1.01 ± 0.19	1.04 ± 0.09^α^
	(0.75–1.29)	(0.84–1.15)	(0.80–1.33)	(0.90–1.18)
LV	1.17 ± 0.09	1.24 ± 0.07^α^	1.20 ± 0.08	1.36 ± 0.11^αβγ^
	(1.03–1.33)	(1.05–1.32)	(1.07–1.34)	(1.20–1.55)
Capillary density (no. mm^−2^)				
SOL	1154.3 ± 175.70	1227.7 ± 174.40	1159.9 ± 187.40	1330.7 ± 166.6^αγ^
	(851.9–1420.7)	(966.6–1465.2)	(919.8–1521.3)	(952.6–1558.8)
PL	930.2 ± 150.4	950.8 ± 199.1	892.0 ± 161.4	1011.1 ± 163.5^γ^
	(707.4–1220.0)	(635.4–1283.2)	(702.1–1145.5)	(705.7–1273.2)
GrL	1119.3 ± 148.9	1249.8 ± 371.3	1185.3 ± 349.6	1317.7 ± 184.2^αγ^
	(912.8–1404.3)	(761.8–2015.2)	(712.7–1860.7)	(961.9–1650.0)
GrM	1061.8 ± 231.3	1193.8 ± 200.9	1063.3 ± 246.9	1180.4 ± 172.7^αγ^
	(751.3–1358.7)	(922.7–1604.4)	(547.7–1439.4)	(947.9–1520.2)
Gw	405.4 ± 79.3	481.1 ± 62.2^α^	441.0 ± 69.9	459.2 ± 57.2^α^
	(249.3–472.8)	(388.5–585.8)	(323.0–540.7)	(377.4–547.7)
LV	2711.9 ± 290.8	2684.4 ± 199.4	2722.6 ± 345.5	2810.1 ± 345.0
	(2235.7–3157.1)	(2235.7–3157.1)	(2185.7–3178.6)	(2271.4–3321.4)

*Note*: Values are presented as means ± SD with range in brackets. Significantly different from the *Sed group using Bayesian data analysis.Τhe 95% confidential interval did not contain the mean value of the ^α^Sed, ^β^ET, and ^γ^HYP groups.

## 4 DISCUSSION

### 4.1 Acute response to exercise under intermittent hypoxia

HIF protein is constitutively expressed in cells, but that is rapidly degraded under normoxic conditions by oxygen‐dependent PHD‐containing enzymes, which have been shown to require oxygen, iron, and 2‐oxyglutarate as co‐factors (Ivan et al., 2001). PHDs are inactivated under hypoxic conditions. Therefore, HIF protein accumulates and translocates to the nucleus, in which it activates HIF‐responsive genes including PHDs, VEGF, erythropoietin, and LDH.

Although total work value during acute exercise and duration of hypoxic exposure in the Int group (Figure S1c) was the same as those in the Alt group (Figure S1b), VEGF mRNA expression levels in Gw were substantially greater in the Int group (CI: 1.19–2.40) than in the Alt group (Figure 2d). Nuclear PHD2 protein levels were significantly upregulated in the Int group but not in the Alt group (Figure 3f). Therefore, HIF activation was facilitated by exercise under intermittent hypoxia, and may persist for at least 3 h after exercise in glycolytic muscle portions. Capillary density in Gw was around one third of that in Gr (Table 4). Therefore, in glycolytic muscle portions, lower capillary supply is probably susceptible to activate HIF during exercise, thereby upregulate VEGFA mRNA levels for a longer period of time compared to slow‐twitch muscle. In rats, nuclear HIF1α protein content was shown to be around three times higher in fast‐twitch extensor digitorum longus muscle than in slow‐twitch SOL muscle (Lunde et al., 2011). Thus, activation of large amount of HIF protein in fast‐twitch muscles may also enable to last higher transcriptional activity levels for a long period of time.

In the Int group, mRNA levels of SIRT6 were also significantly increased in Gw (8.1‐fold, Figure 2f). Thus, upregulated levels of SIRT6 and PHD2 probably suppress transcriptional activity of HIF in glycolytic muscle portions. These results probably indicate that acute exercise under short‐duration intermittent hypoxia enhanced levels of HIF suppressors as well as HIF itself.

### 4.2 Chronic response of exercise training under intermittent hypoxia

The main result of the present study was that endurance exercise training under short‐duration intermittent hypoxic exposure (INT training) facilitated endurance exercise performance via promoting metabolic enzyme activities in left ventricle as well as in hind‐leg muscles.

A rate limiting enzyme of tricarboxylic acid (TCA) cycle, CS activity levels were markedly increased after the INT training in highly oxidative SOL muscle (Figure 4b). Moreover, COX activity levels were substantially enhanced in SOL (CI: 1.01–1.12 vs Sed; CI: 1.05–1.17 vs HYP, Figure 4a). Overexpression of PGC‐1α was shown to enhance CS activity and COX protein levels in rat skeletal muscles (Benton et al., 2010). At 48 h after the last exercise, in SOL, nuclear NT‐PGC‐1α levels were considerably higher in the INT group than in the Sed (1.5‐fold, CI: 1.09–1.79) and ET (1.4‐fold, CI: 1.006–1.66) groups (Figure 8b). Thus, the INT training probably promotes mitochondrial oxidative metabolism via upregulating NT‐PGC‐1α levels in highly oxidative muscle.

In LV, PDHc activity levels were significantly enhanced after the INT training (Figure 6a). HIF1A was shown to upregulate pyruvate dehydrogenase kinase (PDK)‐1, which inhibits PDHc (De Palma et al., 2007). Thus, in the heart, upregulation of PDK1 by HIF may be attenuated during the INT training. After the INT training, MCT4 protein levels were upregulated in LV (Figure 7c). This finding most likely indicates facilitating lactic acid transport toward outside of the cardiac myocytes. Chronic exposure to hypobaric hypoxia (4300 m for 8 weeks) was shown to enhance MCT4 protein levels in the heart (McClelland & Brooks, 2002). As mentioned above, in LV, HIF‐induced PDK1 level is presumably suppressed. Accordingly, the present upregulation of MCT4 is most likely independent of HIF. Thus, the INT training probably promotes pyruvate oxidation and lactate flux in the heart.

Chronic stabilization of HIF was shown to inhibit fatty acid oxidation by reducing PPARα (Belanger et al., 2007) and PGC1α levels in vitro (Slot et al., 2014). The CPT complex facilitates the entry of long‐chain fatty acids from the cytosol into the mitochondrial matrix, in which where beta‐oxidation occurs. Thus, it is considered to be a crucial enzyme of fatty acid utilization. Expression levels of both CPT1 and CPT2 mRNAs were shown to be increased in mice with muscle‐specific overexpression of PGC1α (Cheol et al., 2008). In the present study, CPT2 levels in Gr and PL were significantly greater in the INT group than in the HYP group (Figure 5b). Thus, 4 weeks of the INT training promoted mitochondrial enzyme activities related to fatty acid metabolism in both oxidative and glycolytic muscles. Considerable increases in nuclear NT‐PGC1α levels observed in PL (1.4‐fold, CI: 1.04–1.82 vs Sed) after the INT training may partly explain these adaptive changes (Figure 8b).

LDH enzyme exists in a tetramer formation. Five LDH isozymes are composed of different ratio of the two subunits M and H (Markert, 1963), encoded by the LDHA and LDHB genes, respectively (Stevens & Li, 1989). The H isomer predominantly exists in myocardium and converts lactate to pyruvate in aerobic environments. The M isomer is abundant in skeletal muscles and converts pyruvate to lactate in anaerobic conditions. In contrast to the LDHB gene, the LDHA gene possesses hypoxia recognition sites in its promoter sequence, thereby responsive to HIF (Semenza et al., 1994). Therefore, transcription of the LDHA was shown to be upregulated by acute hypoxia (Firth et al., 1995), whereas the LDHB generally shows no response to hypoxia (Field et al., 1994). In PL, in the present study, LDH‐PL activity values were significantly increased after both HYP and INT training procedures (Figure 6b). Moreover, PDHc activity levels, which was shown to be inhibited by HIF as mentioned above, were considerably decreased after the INT training (CI: 0.93–0.98 vs Sed, Figure 6a) and were negatively correlated with total work values (Table 2). In contrast, nuclear PHD2 levels in PL were considerably increased after the HYP and INT training procedures (Figure 8c). Thus, in PL, HIF may be transiently activated after the HYP and INT exercise regimens, thereby LDHA gene expression and PDHc levels, respectively, were facilitated and inhibited. However upregulated PHD2 inhibited HIF thereafter, thus enzyme activity levels concerning fatty acid oxidation was upregulated (Figure 5).

After the present INT training, nuclear HSF1 levels were considerably increased in SOL (1.6‐fold, CI: 1.004–2.27 vs Sed; 1.7‐fold, CI: 1.02–2.31 vs ET, Figure 8a). HSF1 is a transcription factor that regulates the levels of heat shock proteins in response to various stresses (Zhang et al., 2002). HSF1‐deficiency was shown to delay the regeneration of injured soleus muscle, possibly via a partial depression of increase in Pax7‐positive satellite cells (Nishizawa et al., 2013). In satellite cell‐ablated Pax7 mice, forced daily running of 30 min for 5 days led to a striking loss of myofibers in hind‐leg muscles (Sambasivan et al., 2011). Thus, HSF1 may promote muscle regeneration, thereby induced muscle hypertrophy, identified as increased the relative weight of SOL after the INT training (Table 1). In HSF1 deficient mice, exercise‐induced capillary growth was shown to be suppressed by downregulation of HIF and VEGF levels in pressure overloaded heart (Tian et al., 2020). Thus, HSF1 may also contribute to capillary growth observed in SOL after the INT training (Table 4).

The HYP training, in the present study, increased exercise performance, but less extent than the INT training did. After the HYP training, in Gw, activity levels of COX (Figure 7a), CS (Figure 4b), and LDH‐LP (Figure 6c) were significantly increased and levels of FABP were considerably upregulated (2.3‐fold, CI: 1.48–3.21 vs Sed, Figure 7a). Furthermore, in PL, activity levels of HAD (Figure 5a) and LDH‐PL (Figure 7b) were significantly increased after the HYP training. These findings probably indicate that the HYP training facilitates exercise performance via promoting lactate flux, oxidative phosphorylation, and fatty acid utilization in highly‐glycolytic muscles. Moreover, the HYP training facilitated fatty acid metabolism in DIA, identified by significant increase in CPT2 activity levels (Figure 5b), suggesting that HYP training probably facilitates fatty acid utilization in respiratory muscles. This finding also presumably contributes to promoting endurance performance.

In conclusion, for the first time, the present study demonstrated that the INT training, exercise training under short‐duration intermittent hypoxia every other day for 4 weeks, clearly had additive effects on training‐induced increases in endurance performance. The INT training promoted enzyme activity levels in a rate limiting enzyme of TCA cycle in several skeletal muscle probably via upregulating nuclear NT‐PGC1α protein levels. Furthermore, it facilitated enzyme activity levels concerning pyruvate oxidation and protein levels of lactate transporter in the heart. The INT training also promoted capillary growth in skeletal muscle and heart. The present findings show that the INT training represents a beneficial strategy for increasing endurance performance. To identify the INT training has prominent effects on endurance performance for the athletes, some fundamental experiments should be done with well‐trained animals.

## AUTHOR CONTRIBUTIONS

The author J.S. was involved in the conception and design of the study, analyzing the data, and preparing the first draft of the manuscript. The author revised the draft manuscript and approved the final concept. The author agrees to be accountable for all aspects of the work in ensuring questions relating to the accuracy and integrity of any part of the work are appropriately investigated and resolved.

## FUNDING INFORMATION

This work was supported by JSPS KAKENHI Grant Number 20K11410.

## CONFLICT OF INTEREST

None declared.

## ETHICS STATEMENT

All procedures were approved by the Animal Care and Use Committee of Hokkaido University of Education (No. 7, approved on 2021/4/20) and performed in accordance with the “Guiding Principles for the Care and Use of Animals in the Field of Physiological Sciences” of the Physiological Society of Japan.

## Supporting information


Figure S1.

Figure S2.

Figure S3.
