# Supplementary figures and images for: Endurance exercise under short‐duration intermittent hypoxia promotes endurance performance via improving muscle metabolic properties in mice
Source: Physiol Rep. 2022 Dec 14;10(23):e15534. doi: 10.14814/phy2.15534 (PMC9748492; doi:10.14814/phy2.15534)

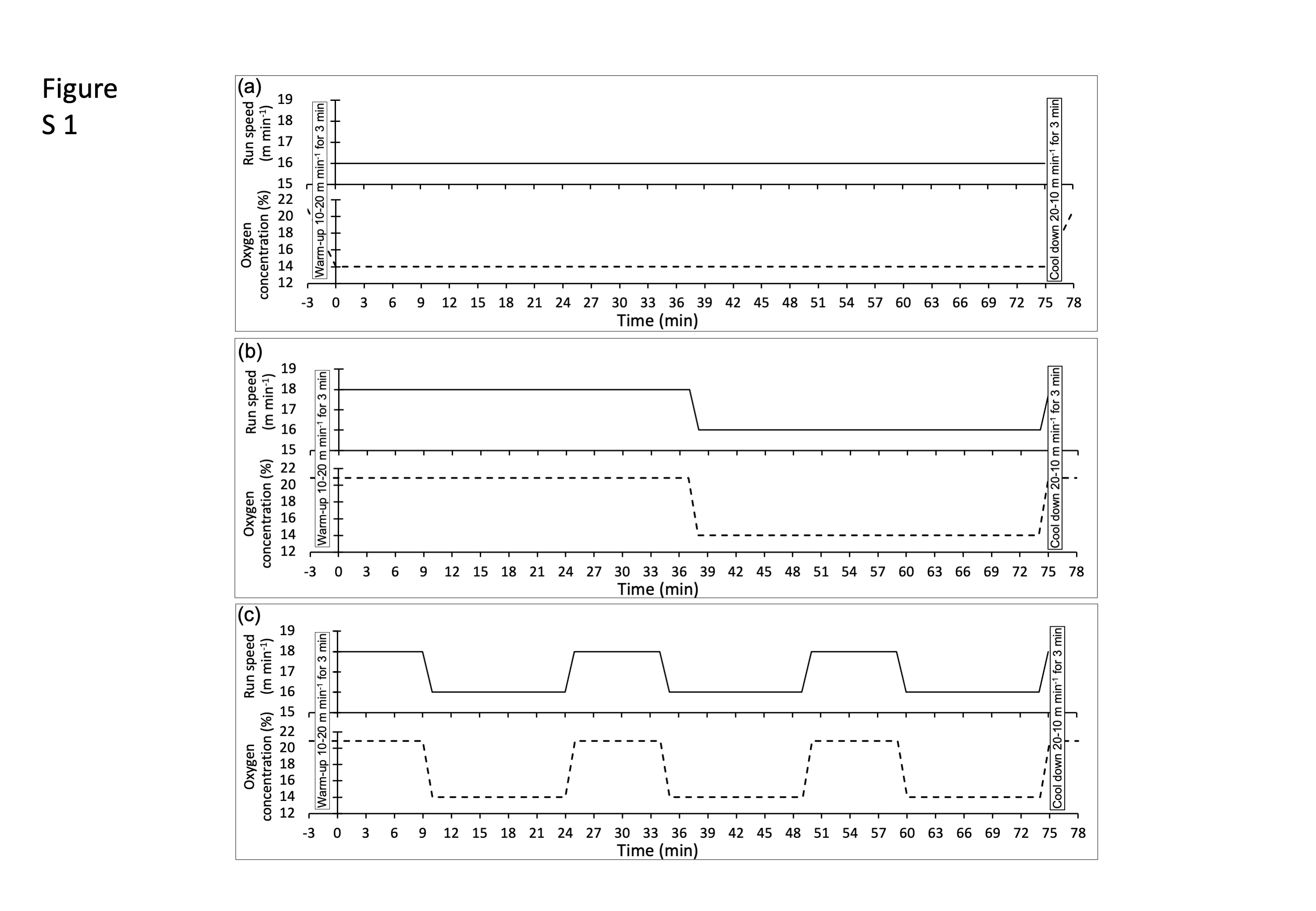

Supplement: Supplementary file 1 — Figure S1. [file PHY2-10-e15534-s002.tiff]

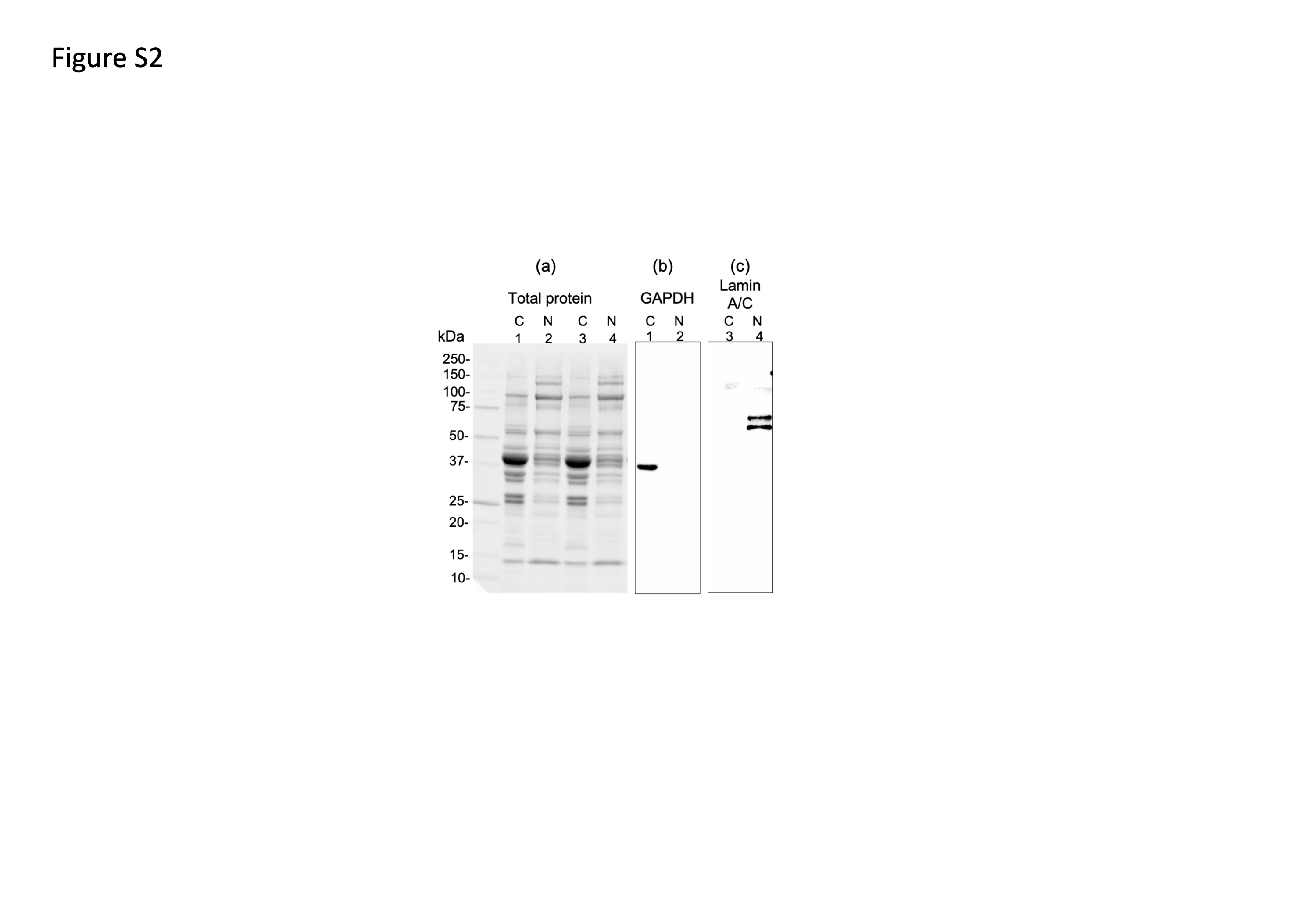

Supplement: Supplementary file 2 — Figure S2. [file PHY2-10-e15534-s003.tiff]

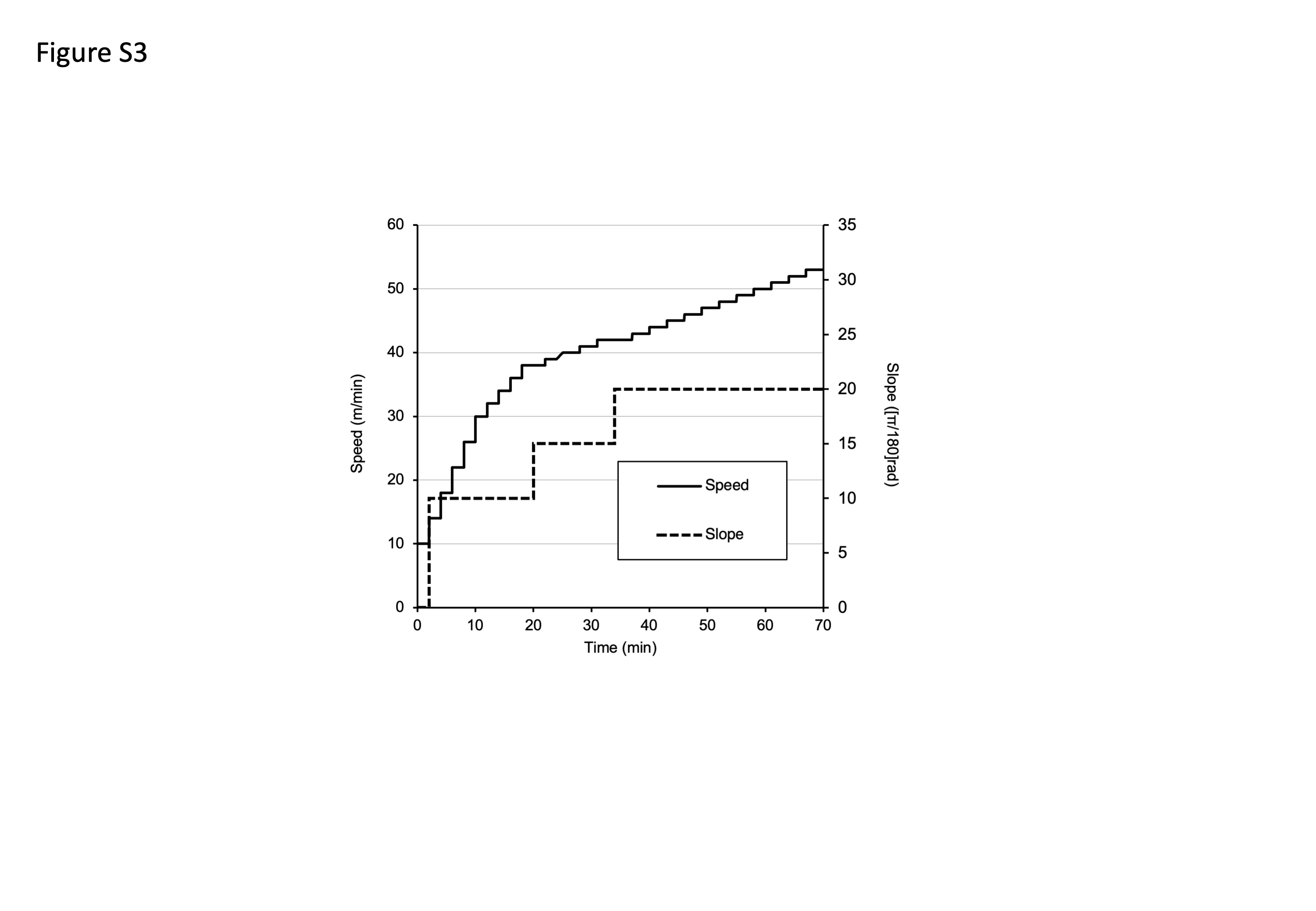

Supplement: Supplementary file 3 — Figure S3. [file PHY2-10-e15534-s001.tiff]
